# Moderating Role of Resilience Between Depression and Stress Response of Vocational Middle School Students During the COVID-19 Pandemic

**DOI:** 10.3389/fpsyt.2022.904592

**Published:** 2022-07-01

**Authors:** Mingqi Jin, Lingling Ding, Jiali Fan, Xin Sheng, Bingqing Luo, Ronghua Hang, Linpu Feng, Long Huang

**Affiliations:** ^1^School of Humanities and Management, Wannan Medical College, Wuhu, China; ^2^School of Education Science, Nanjing Normal University, Nanjing, China

**Keywords:** resilience, depression, stress response, vocational middle school students, the COVID-19 pandemic

## Abstract

**Object:**

In this study, we aimed to explore the influences of stress responses and psychological resilience on depression of vocational middle school students during the initial COVID-19 outbreak in China.

**Methods:**

An online questionnaire survey on the students of a medical school in Jiangxi Province, China, and obtained 3,532 valid questionnaires. A self-compiled general situation questionnaire, Stress Response of COVID-19 Questionnaire, the Resilience Scale for Chinese Adolescents and Center for Epidemiological Studies Depression Scale (CES-D) were used. Hierarchical regression analysis was used to explore the regulatory role of psychological resilience between stress response and depression.

**Results:**

(1) There were significant differences in gender between vocational middle school students' evaluation (t = 3.07, *P* = 0.002) and defense (t = 3.28, *P* = 0.001) of the pandemic. Males had higher cognitive evaluation of the pandemic than females, and females had more defense against the pandemic than males. (2) There is a significant difference between vocational middle school students from different grades in depression level (F = 3.62, *P* = 0.03), pneumonia defense (F = 13.65, *P* < 0.001) and pneumonia panic (F = 3.10, *P* = 0.045). (3) Depression level (F = 7.17, *P* < 0.001), pneumonia evaluation (F = 2.78, *P* = 0.04) and pneumonia panic (F = 3.32, *P* = 0.02) of the students concerning the spatial distance of the pandemic. (4) The severity of urban pandemic affects the evaluation of pneumonia among vocational middle school students. (5) Depression was negatively correlated with psychological resilience and pneumonia evaluation, and positively correlated with pneumonia panic. Psychological resilience was positively correlated with pneumonia evaluation and pneumonia defense, and negatively correlated with pneumonia panic. (6) Psychological resilience could reduce the level of depression caused by pneumonia evaluation and pneumonia panic.

**Conclusion:**

There were significant differences in depression level and stress responses in grades, gender and spatial distance of pandemic. Resilience has a significant negative moderator effect on the relationship between pandemic panic and depression. Resilience has a significant positive moderator effect on the relationship between pandemic evaluation and depression.

## Introduction

The novel coronavirus pandemic (COVID-19), which began in December 2019, is a global public health emergency, and its impact has continued to this day. As the virus mutates rapidly, spreads rapidly and may bring adverse consequences such as health problems, it brings anxiety and panic to people. In order to deal with the spread of the virus, various countries and regions have launched different response strategies, such as reducing aggregation, lockdown of the cities, shutdown or home office, school suspension or e-learning. Although these measures are designed to protect people from the virus, they also bring new problems. The fear of infection and the disruption of routine life lead to the decline of people's mental health, and the incidence rate of psychological problems such as depression, anxiety, loneliness, substance abuse, suicide risk, sleep and eating disorders are significantly higher compared with the situation before the pandemic (1).

Teenagers have been paid special attention in the research of people's mental health during the pandemic. Like everyone else, they are worried that they and their families will be infected by the virus and that they will infect others without their knowledge (2). In the worst period of the pandemic, they need to temporarily stop the traditional face-to-face learning and rely more on online distance learning. They are far away from campus and peers, and their normal social communication is affected. Since the adolescents are not mature enough, their mental health is affected in some way and the incidence of various psychological disorders is higher than that before the pandemic when they are faced with the pressure of pandemic, isolated family life and learning style (3–5). In different research reports, the positive screening rate of adolescent depression reaches 20–40%. Anxiety and other stress-related psychological disorders also increased significantly more than other age groups (6–8). In addition to the above reasons, the pressure facing junior high school students and senior high school students also makes their levels of depression and anxiety higher. One way to help them reduce their levels of depression and anxiety is to improve their resilience.

Psychological resilience usually refers to the ability of individuals to mobilize personal resources (protective factors) to maintain or quickly recover normal psychological functions after experiencing adversities or traumas. It is a successful response to the “self-adjustment mechanism”. It is found that individuals with high resilience perceive less psychological distress and have a higher level of mental health than those with low resilience (9). During the new coronavirus pandemic, a survey of 3,042 subjects showed that individuals with high resilience had lower levels of anxiety and depression than those with low resilience. In the youth group, affected by learning pressure, parent-child relationship, cognitive limitations and lack of experience, their psychological resilience is often less than that of adults (2).

In the study of teenagers, junior high school students and senior high school students are the objects of more attention, but the research of other adolescent groups is relatively scarce, such as secondary vocational school students in this study. These students enter vocational schools after graduating from junior high school. They usually will graduate and start working after 3–5 years of study. Compared with high school students of the same age, they generally do not face the pressure of entering a higher school, so they have less pressure, but they will have the pressure of employment. In this study, our respondents came from a secondary occupational vocational middle school (hereinafter referred to as vocational middle school). Most of the students in this school are nursing majors, and others are medical related majors. This means that they have to face not only the pressure of employment, but also the possibility of close contact with the pandemic as medical workers.

The purpose of this study is to understand the current situation of stress response and depression of vocational middle school students during the pandemic of COVID-19 through questionnaire survey, and to explore the impact of COVID-19 stress response on depression, as well as the regulatory role of psychological resilience between COVID-19 stress response and depression. In addition, we will also analyze the effects of variables such as the severity of COVID-19 and spatial distance on depression.

Accordingly, we simulated the relationship model between stress response and depression and proposed the following hypothesis:

Hypothesis 1: The stress response of COVID-19 will lead to the depression of vocational middle school students.

Hypothesis 2: Psychological resilience plays a regulatory role between stress response and depression during the COVID-19 pandemic.

## Methods

### Study Population and Sample

In this study, the students of a secondary occupational vocational middle school were investigated by anonymous cluster sampling through the Internet in Jiangxi Province, China. The survey was carried out in April 2020. A total of 4,074 questionnaires were distributed, and 3,532 valid questionnaires were recovered, with an effective recovery rate of 86.25%. There were 379 males and 3,153 females. The average age was 17.03 ± 1.29 years.

### Rating Instruments

#### Self-Compiled General Situation Questionnaire

Including gender, age, grade, major, city (determine the severity of urban pandemic according to the number of infected people in the city, 1 = infected people <50, 2 = infected people 51–100, 3 = infected people 101–150, 4 = infected people more than 150), the infection situation of COVID-19 in the district/administrative village (determine the spatial distance between COVID-19 and the participants according to this, 0 = unaffected, 1 = home isolators, 2 = suspected patients, 3 = confirmed patients).

#### Stress Response of COVID-19 Questionnaire

Revised according to the SARS Stress Response Questionnaire compiled by Tong (10). The Stress Response of COVID-19 Questionnaire was revised, with 13 questions in total. And three contents are measured: pandemic evaluation, pandemic panic, pandemic defense. Cronbach's α coefficients of pandemic evaluation, pandemic panic, and pandemic defense were 0.62, 0.73, and 0.66 respectively.

#### The Resilience Scale for Chinese Adolescents

The Resilience Scale compiled by Hu and Gan (11) was used to measure the resilience level of vocational health school students. There are 27 questions in the scale, which are scored by 5 points. The scale is divided into two dimensions: individual manpower and support, including 15 questions for individual manpower and 12 questions for support. The higher the total average score, the higher the Resilience. In this study, Cronbach's α coefficient is 0.89.

### Center for Epidemiological Studies Depression Scale

Using the Center for Epidemiological Studies Depression Scale (CES-D) compiled by Radloff (12), there are 20 items in the scale, which are graded from 0 to 3, among which 4 items are reverse scores. The sum of scores of all items is the total score of the scale. If the total score is less than or equal to 15, it indicates that there is no depression. Depression may be judged when the score is 16–19. If the total score is greater than or equal to 20, it indicates that there is depression. The higher the score, the more severe the degree of depression. In this study, the Cronbach's α coefficient is 0.87.

### Statistical Analyses

SPSS22.0 was used to collate and analyze the data. The data were descriptively analyzed by using mean and standard deviation. Independent sample *t*-test and one-way ANOVA were used to compare the effects of gender, grade, severity of urban pandemic and spatial distance of pandemic on stress response to COVID-19 and depression level. Correlation analysis was used to explore the relationship between stress response, depression and psychological resilience. Hierarchical regression analysis was used to explore the regulatory role of psychological resilience between stress response and depression.

## Result

### Stress Response, Resilience and Depression

The positive rate of depression screening was 19.4% in our study. Unpaired *T*-test and one-way ANOVA were used to analyze the influencing factors of depression and stress response of vocational middle school students. The results are shown in Table 1. The evaluation of pandemic situation (t = 3.07, *P* = 0.002) and defense (t = 3.28, *P* = 0.001) were significantly different in gender, while the depression level of vocational middle school students was borderline significant in gender. Compared with females, males have a higher cognitive evaluation of the pandemic situation, and females have more defensive and depression than males. There were significant differences in depression level (F = 3.62, *P* = 0.03), pandemic defense (F = 13.65, *P* < 0.001)and pandemic panic (F = 3.10, *P* = 0.045)in different grades. There were differences in the severity of urban pandemic. There are significant differences in depression level (F = 7.17, *P* < 0.001), pandemic panic (F = 3.32, *P* = 0.02)and pandemic evaluation (F = 2.78,*P* = 0.04) among spatial distance of pandemic.

**Table 1:** The mean score and standard deviation of depression and stress response.

**Variable**		**Depression**			**Pandemic evaluation**			**Pandemic panic**			**Pandemic defense**		
		**M ±SD**	**F/t**	**p**	**M ±SD**	**F/t**	**p**	**M ±SD**	**F/t**	**p**	**M ±SD**	**F/t**	**p**
Gender	Male	9.62 ± 7.86	−1.67	0.095	9.88 ± 1.75	3.07	0.002	6.28 ± 2.33	1.57	0.116	13.88 ± 3.07	−3.28	0.001
	Female	10.34 ± 8.01			9.60 ± 1.708			6.09 ± 1.75			14.42 ± 2.71		
Grades	1	10.71 ± 8.16	3.62	0.027	9.55 ± 1.74	2.11	0.120	6.02 ± 1.76	3.10	0.045	14.19 ± 2.84	13.65	0.000
	2	10.10 ± 7.80			9.67 ± 1.75			6.12 ± 1.86			14.22 ± 2.71		
	3	9.87 ± 7.95			9.68 ± 1.65			6.21 ± 1.85			14.73 ± 2.66		
Severity of urban pandemic	1	10.45 ± 7.99	1.54	0.203	9.55 ± 1.70	7.38	0.000	6.06 ± 1.90	0.26	0.852	14.39 ± 2.68	0.60	0.604
	2	10.54 ± 7.98			9.69 ± 1.68			6.10 ± 1.78			14.48 ± 2.75		
	3	10.63 ± 7.72			9.37 ± 1.81			6.16 ± 1.82			14.29 ± 2.80		
	4	9.98 ± 8.09			9.72 ± 1.68			6.11 ± 1.82			14.33 ± 2.76		
Spatial distance of pandemic	0	9.98 ± 7.82	7.17	0.000	9.66 ± 1.72	2.78	0.040	6.08 ± 1.81	3.32	0.019	14.31 ± 2.75	2.33	0.072
	1	11.51 ± 7.59			9.30 ± 1.81			6.16 ± 2.01			14.58 ± 2.94		
	2	11.62 ± 8.38			9.44 ± 1.59			6.47 ± 1.98			14.74 ± 2.78		
	3	11.67 ± 9.3			9.88 ± 1.66			6.14 ± 1.71			14.51 ± 2.69		

### Correlation Between Continuous Variables

After controlling gender and age, the descriptive statistics and correlation analysis are shown in Table 2. The results show that depression is negatively correlated with resilience and pandemic evaluation, and positively correlated with pandemic panic. Resilience was positively correlated with pandemic evaluation and pandemic defense, but negatively correlated with pandemic panic. In the stress response of COVID-19 subscale, pandemic evaluation was negatively correlated with pandemic panic and positively correlated with pandemic defense.

**Table 2:** Correlation analysis of depression, resilience and stress response.

	**M**	**SD**	**1**	**2**	**3**	**4**	**5**
1. Evaluation	9.63	1.72	1.00				
2. Panic	6.11	1.82	−0.07^***^	1.00			
3. Defense	14.36	2.75	0.09^***^	0.35^***^	1.00		
4. Depression	10.26	7.99	−0.18^***^	0.38^***^	0.01	1.00	
5. Resilience	3.37	0.53	0.17^***^	−0.15^***^	0.06^***^	−0.50^***^	1.00

*** *p < 0.001*.

### Hierarchical Regression Analysis

According to Wen et al. (13), the moderator effect of resilience on stress response of COVID-19 and depression was investigated. The regression analysis results are shown in Table 3. Pandemic evaluation, pandemic defense and resilience can significantly negatively predict depression. Pandemic panic can positively predict depression. At the same time, resilience has a significant negative moderator effect on the relationship between pandemic panic and depression. Resilience has a significant positive moderator effect on the relationship between pandemic evaluation and depression. Therefore, resilience has moderator effect on the relationship between pandemic defense and depression.

**Table 3:** Regression analysis of depression.

	**Equation 1 (variable: depression)**		**Equation 2 (variable: depression)**		**Equation 3 (variable: depression)**	
	**β**	**t**	**β**	**t**	**β**	**t**
Spatial distance	0.074	4.408^***^	0.061	3.974^**^	0.043	3.234^**^
Pandemic evaluation			−0.145	−9.450^***^	−0.450	−5.147^***^
Pandemic panic			0.405	24.797^***^	1.072	11.960^***^
Pandemic defense			−0.117	−7.167^***^	−0.204	−2.197^*^
Resilience					−0.501	−4.634^***^
Pandemic evaluation × resilience					0.547	4.258^***^
Pandemic panic × resilience					−0.805	−8.429^***^
Pandemic defense × resilience					0.201	1.622
R^2^	0.005^***^		0.181^***^		0.370^***^	
ΔR^2^	0.005^***^		0.177^***^		0.190^***^	
F	19.427^***^		195.958^***^		260.042^***^	

* *p < 0.05*,

** *p < 0.01*,

*** *p < 0.001*.

## Discussion

During the pandemic period, the overall depression level of the subjects in this study was lower than that of other people of the same age group in the same period (5). Due to the lack of mental health data of this group before the outbreak, we speculate that the reason for this result may be related to the survey time, the method of using online survey, the limitations of sampling and the fact that the respondents are not in high-risk areas. Because in other surveys with more extensive sampling, even among senior medical students who have more knowledge reserves and clinical experience, the positive screening rate of depression during the pandemic is still as high as 24.3%, which is 70% higher than that before the pandemic (5). The sampling time of this survey is April 2020. At this time, the condition of the pandemic in China has been in the stage of steady decline, and Jiangxi Province, where the medical school is located, is not a serious pandemic area. These may be the reasons why the positive screening rate of depression is lower than the average level. Because in the same period, the results of a repeated survey on the pandemic emotional state of young people in the United States showed that the positive rate of depression among the respondents was quite high in May 2020, but it also decreased 1 month later (14).

Gender is one of the important factors affecting depression (15, 16). Other studies have similar findings, that is, the level of depression in female is higher than that in males, and the difference is significant, which may be related to women's strong risk perception. It has been found that during the outbreak of 2019 coronavirus disease, the perceived risk and severity of the virus are related to poor mental health (17). The study of 2019 coronavirus disease found that women spend more time thinking about COVID-19 than men every day, and have more depressive symptoms than men. In addition, in the cognitive evaluation of influenza 2019 coronavirus disease, males' COVID-19 cognitive score is higher than that of female, and females' score in pandemic defense is higher than that of males. The reason may be that men have a better understanding of 2019 coronavirus disease, which leads to the reduction of depression. However, women's cognitive evaluation of 2019 coronavirus disease is comparatively lower, and this consequently leads to the aggravation of depression. However, female will also take more defensive measures to help themselves reduce their emotional distress. This result explains the relationship between gender, depression and stress response to some extent. Some studies have reached similar conclusions. They found that women tend to take defensive actions when coronavirus disease occurs in 2019 (18, 19), so they are more willing to follow prevention and control recommendations, such as wearing masks and avoiding going to public places (20).

This survey shows that the severity of urban pandemic affects pandemic evaluation, but did not affect pandemic panic and pandemic defense, and had no significant difference in depression. This shows that although the severity of COVID-19 pandemic is different in different cities, there is no obvious difference in the effects on panic, defense and depression of vocational middle school students. This is consistent with the research results of Huang et al. (21) on nursing students and nurses, and their research conclusions also show that the severity of urban pandemic has no significant impact on the subjects' emotions and coping styles. The reason for this result may be that in our survey sample, even in those cities with severe COVID-19, the number of confirmed cases in COVID-19 is not much, only 217 cases at most. In the case of great differences in the pandemic situation in COVID-19, whether it is a cross-provincial and cross-regional comparative study or a comparison of different countries (the United States and Israel), it is found that the depression and anxiety of people in countries or regions with severe pandemic situation are significantly higher than those in countries or regions with mild pandemic situation, and the difference is significant (2). Distance has significant influence on depression, pandemic evaluation and panic of vocational middle school students. On the whole, the closer COVID-19 is to an individual (i.e., when there are infected people in the community or village), the higher the level of depression and panic of the individual, that is, the presence of confirmed cases of COVID-19 near the individual's residence will lead to more tension and emotional and behavioral problems, which is consistent with the research results of Huang et al. (22).

The results of correlation analysis showed that resilience was negatively correlated with pandemic panic and depression, and positively correlated with pandemic evaluation and pandemic defense. Further regression analysis showed that pandemic evaluation, pandemic defense and pandemic panic can directly predict depression, and pandemic evaluation and pandemic panic can also adjust depression level through resilience. From the difference between the weak resilience group and the strong resilience group, it can be concluded that even if the cognitive evaluation of pandemic is low and the degree of panic is high, the high resilience can reduce the depression and relieve the negative emotional reaction caused by stress (6). In the research of various catastrophic events, resilience has been found to protect individual mental health and help people cope with disasters and tide over crises (23, 24). Among the survivors of the earthquake, tsunami and nuclear disaster in Japan, higher resilience indicates lower level of post-traumatic stress disorder and depressive symptoms. After Wenchuan earthquake, the same group of subjects were followed up three times in 6 months, 18 months and 24 months, which showed that resilience played an important role in the post-traumatic growth of individuals after the earthquake (25). In the COVID-19, adolescents' psychological resilience is affected by factors such as active coping, social support, family economy and parents' education level. Adolescents with high psychological resilience have a more stable psychological state and a higher level of mental health when coping with this stress event (24). Another survey of medical staff found that improving sleep quality and life satisfaction can improve the psychological resilience of medical staff and increase their ability to deal with risks (26). Therefore, good resilience not only means survival and adaptation to challenges, but also indicates growth, development and better after crisis events (27).

In conclusion, improving resilience can help individuals recover from normal life faster, reduce the occurrence of depression or alleviate depressive symptoms. Through the research and comprehensive analysis of vocational middle school students, we believe that during the period of pandemic isolation at home, it can improve the individual's ability to deal with negative life events by improving the individual's awareness of pneumonia by imparting correct knowledge, enhancing the individual's defense ability against pneumonia, and reducing the psychological panic of pneumonia through psychological regulation and social security. The individual can still maintain adequate adaptability and reduce the level of depression during stressful events (28). Other studies have found that the impact of psychological and social factors on negative emotions may be greater than the direct impact of the virus itself on individuals, so healthy diet, regular exercise and appropriate emotional connection with others all help to increase resilience (29, 30). For groups with high levels of depression, such as women, young people and people with low educational background, support services and activities to strengthen mental health can be provided in a planned way (31).

## Limitations

Although we have obtained some meaningful results through research and analysis, there are still some deficiencies in this study. Firstly, as a cross-sectional study, it is difficult to clarify the causal relationship between variables, which needs to be supplemented by necessary longitudinal research. Secondly, the homogeneity of the sample is high, and the imbalance between men and women may lead to weak representativeness of the results and large statistical errors. Therefore, it is necessary to increase the data of different groups and improve the popularization value of the results. Thirdly, the self-report form used in the survey is also easy to lead to the deviation of the results. Despite the above limitations, this study managed to find out the probability of depression and its influencing factors by exploring the relationship between psychological variables. These findings will help to provide reference results for future research and provide data support for relevant departments and scholars to take targeted psychological intervention measures.

## Conclusion

Compared with females, males have a higher cognitive evaluation of the pandemic situation, and females have more defensive and depression than males. There were significant differences in depression level, pandemic defense and pandemic panic in different grades. There were differences in the severity of urban pandemic. There are significant differences in pandemic level, pandemic panic and pandemic evaluation among spatial distance of pandemic. Resilience has a significant negative moderator effect on the relationship between pandemic panic and depression. Resilience has a significant positive moderator effect on the relationship between pandemic evaluation and depression.

## Data Availability Statement

The raw data supporting the conclusions of this article will be made available by the authors, without undue reservation.

## Ethics Statement

The studies involving human participants were reviewed and approved by Academic Ethics Committee of Wannan Medical College. Written informed consent to participate in this study was provided by the participants' legal guardian/next of kin.

## Author Contributions

LH and LF conceived and designed the review. MJ and LL wrote the manuscript. LH analyzed the data. HR, LL, LJ, BL, and XS revised the manuscript. All authors contributed to the article and approved the submitted version.

## Funding

This study was supported by the Outstanding Young Talent Support Program Project of Anhui Province (Grants No. gxyq2020160), the University Science Research Project of Anhui Provincial Department of Education (Grants No. SK2020ZD33), and the Philosophy and Social Science Planning Project of Anhui Provincial (Grants Nos. AHSKQ2020D129 and AHSKF2021D21).

## Conflict of Interest

The authors declare that the research was conducted in the absence of any commercial or financial relationships that could be construed as a potential conflict of interest.

## Publisher's Note

All claims expressed in this article are solely those of the authors and do not necessarily represent those of their affiliated organizations, or those of the publisher, the editors and the reviewers. Any product that may be evaluated in this article, or claim that may be made by its manufacturer, is not guaranteed or endorsed by the publisher.
